# Supplementary material for: A qualitative study of the barriers to using blinding in in vivo experiments and suggestions for improvement
Source: PLoS Biol. 2022 Nov 17;20(11):e3001873. doi: 10.1371/journal.pbio.3001873 (PMC9714947; doi:10.1371/journal.pbio.3001873)
Supplement: S1 Table — The term blinding is used, as this was the terminology used during the interviews. (DOCX) [file pbio.3001873.s001.docx]

**Supplementary Table 1:** Interview guide questions. The term blinding is used, as this was the terminology used during the interview.

| **Area explored** | **Type of questions** |
| --- | --- |
| Overview of experiment | Species  Objective(s)/outcomes  Description |
| Was blinding used? | Who was blinded? To what? (and who was involved)  At what point in the process was this done?  When was the experimenter “de-blinded”? |
| How was allocation done? | How was this recorded?  Who could access it? |
| Experimental set-up relevant to randomisation and/or blinding | How are subjects identified?  How is welfare monitoring done?  What software was used to collect data? (how automated is the process?) |
| Other barriers? | What barriers (practical/staff/policy) have you encountered to blinding before allocation? during the experiment? at point of measurement? during analysis?  How could these be overcome?  What would need to change?  Would another pair of hands help? Who could this be? |
| Anything out of your control that would need to change? | For example, policy at the animal unit |
| Any other thoughts on blinding? |  |
