# Supplementary material for: A qualitative study of the barriers to using blinding in in vivo experiments and suggestions for improvement
Source: PLoS Biol. 2022 Nov 17;20(11):e3001873. doi: 10.1371/journal.pbio.3001873 (PMC9714947; doi:10.1371/journal.pbio.3001873)
Supplement: S2 Table — The table presents the data collected during the interview, including the randomisation and masking status for each step of the experiment, and the barriers identified for each study. In bold, we indicate the coding assigned to each barrier during our analysis. These in-house rodent experiments, conducted within the UK, assessed either the efficacy, toxicity, or mechanism of action for a pharmaceutical intervention (e.g., drug treatment). As the experiment type was conducted by multiple people, the answers were obtained from group interviews and represents the typical setup within the company. As some issues are common across experiments and arising from the framework within which the studies are conducted, these have been grouped together for conciseness. The codes assigned to a particular study are not a fixed attribute of that study type but reflect the way these experiments were conducted. There were 8 interview groups where the number of researchers in each group varied from 1 to 3. No researcher sat on multiple groups. Which group discussed which studies are captured in the study type column. (DOCX) [file pbio.3001873.s002.docx]

**Supplementary Table 2:**

Results and analysis of the interviews conducted at a pharmaceutical company during 2018. The table presents the data collected during the interview, including the randomisation and masking status for each step of the experiment, and the barriers identified for each study. In bold we indicate the coding assigned to each barrier during our analysis. These in-house rodent experiments, conducted within the UK, assessed either the efficacy, toxicity or mechanism of action for a pharmaceutical intervention (e.g., drug treatment). As the experiment type was conducted by multiple people, the answers were obtained from group interviews and represents the typical set-up within the company. As some issues are common across experiments and arising from the framework within which the studies are conducted, these have been grouped together for conciseness. The codes assigned to a particular study are not a fixed attribute of that study type but reflect the way these experiments were conducted. There were 8 interview groups where the number of researchers in each group varied from 1 to 3. No researcher sat on multiple groups. Which group discussed which studies are captured in the study type column.

| **Study type** | **How is randomisation implemented during the study?** | **How is masking implemented during allocation and intervention?** | **How is masking implemented during the conduct of the experiment?** | **How is masking implemented during the outcome assessment?** | **How is masking implemented during data analysis?** |
| --- | --- | --- | --- | --- | --- |
| New model tumour growth studies  (Group A, N=2) | If multiple compounds or conditions are being tested in one experimental run, then animals were allocated to experimental group using a randomisation tool based on body weight. | Not required: no control group and results are descriptive. | Not required: no control group and results are descriptive. | Not required: no control group and results are descriptive. | Not required: no control group and results are descriptive. |
| Tolerability studies  (Group B, N=2) |  |  |  |  |  |
| Pharmacokinetic (PK) studies  (Group A, N=2) |  |  |  |  |  |
| Safety telemetry studies  (Group C, N=3) | Not applicable as there is only one group of animals | Not required: an escalating dose design | Not required: an escalating dose design | Not required: an escalating dose design | Independent analysis (statistician). However, no formal masking strategies were then implemented.  **Knowledge constraint:** aware of the concept of masking but unaware of masking strategies  **Culture constraint**: not the norm – no one does it. |
| Anti-tumour studies (small molecules)  (Group D, N=2) | Animals were allocated to experimental group using a randomisation tool based on body weight or other covariate considered important to the experiment. | For most studies, support staff who are not invested in the outcome operating in a high throughput environment conduct the allocation and dosing.  A **practical constraint** to full masking arises as the animals are housed in experimental groups due to coprophagia and a **fear or errors** (dosing mistakes). The intervention information was shown on the cage card and on the IT collection pages **(technological &** **operational constraints**). **Welfare concerns** require rapid identification of what an individual animal has received, and the software therefore showed this information on most pages introducing a **technological constraint** to masking. | For most studies support staff, who are not invested in the outcome, conduct the day-to-day animal care. A **practical constraint** to full masking is that animals are housed in experimental groups due to coprophagia and **fear of errors** (dosing mistakes). Furthermore, the intervention information is shown on the cage card (**operational constraint**) because of a **welfare concern.** There is a requirement to rapidly identify what an individual animal has received, and the software therefore shows this information on most pages introducing a **technological**  **constraint** to masking.  Culture of differential animal care requiring knowledge of intervention. | Support staff, who are not invested in the outcome and operate in a high throughput environment, conduct the measurements.  Dosing and measurement happen simultaneously to minimize stress by reducing the handling of animals.  Intervention information was known due to  **Practical, technological, operational and welfare constraints** (see info from allocation section)**.** | Not implemented  **Resource constraint:**  only one person conducting the entire experiment who then analysed the data.  **Knowledge constraint:**  lack of awareness of strategies on how masking is implemented in practice.  -Lack of awareness of value of masking during analysis.  **Culture constraint**: nobody masks during analysis  **Belief in the value**: questioned value of masking when it adds more work.    **Fear of errors**: it would be complex to implement and mistakes could be made |
| Anti-tumour studies (large molecules)  (Group E, N=2) |  | For most studies support staff, who are not invested in the outcome and operate in a high throughput environment, conduct the allocation and dosing.  Animals were housed in experimental groups due to coprophagia (**practical constraint**) and **fear of errors** (dosing mistakes) and hence labelled with experimental group ID but not intervention information. **Welfare concerns** required rapid identification of what an individual animal had received, therefore this information is shown on a separate page that can be accessed if required. |  | Support staff, who are not invested in outcome operating in a high throughput environment, conduct the measurements using an automated digital calliper system which uses a chip in the flank to identify the animals.  During the data collection, intervention information was hidden but can be accessed if required. |  |
| Biomarker studies – *ex vivo* (oncology)  (Group F, N=2) |  |  |  | Samples labelled with animal ID and experimental group ID. Associated documentation shows the intervention (**operational constraint**). Samples are typically processed by the scientist who initiated the study.  **Resource constraint:**  easier to prioritise and implement complex experiment with a single scientist  **Knowledge constraint:**  lack of awareness that you should mask when processing a sample. Thought it was only for qualitative assessments.    **Culture constraint:** we always label our samples clearly.  **Belief in the value**: questions were raised as to the value when it adds more work.  **Fear of errors:** was expressed as worries over mix up of samples if masking was implemented.  **Ownership issues**: did not want to involve others as who conducts the work leads to recognition. |  |
| IRWIN assay  (Group C, N=3) |  | For most studies support staff, who are not invested in the outcome, conduct the allocation and dosing.  A **practical constraint** to masking is that animals are housed in experimental groups due to coprophagia and a **fear of errors** (dosing mistakes) such that intervention information is shown on the cage card (**operational constraint**). **Welfare concerns** require rapid identification of what an individual animal has received, and the software therefore shows this information on most pages introducing **technological & operational constraints** to implementing masking. |  | Support staff are utilized to enable masked assessment of an individual animal during qualitative assessment. |  |
| Grip strength  (Group C, N=3) |  |  |  | Support staff are utilized to enable masked assessment of an individual animal during testing. |  |
| Rotarod  (Group C, N=3) |  |  |  | Not implemented  An automated testing system that tests animals from multiple experimental groups simultaneously (block design) is used.  **Technological constraint:** staff can see the intervention information through the IT systems.  **Belief in the value:** did not consider that masking would improve the results |  |
| PICA studies  (Group C, N=3) |  |  |  | Samples are collected by support staff and labelled with animal ID. This masks the investigator during quantification to the intervention information. |  |
| Optometry  (Group C, N=3) |  |  |  | Not implemented  **Resource constraint:** the investigator works alone and can see the intervention information from both the cage cards and the IT systems during data submission (**technological & operational constraints**). |  |
| Allergen challenge – ear/paw thickness assessment  (Group G, N=3) |  |  |  | Support staff are utilized to enable masked assessment of an individual animal during qualitative assessment. |  |
| Allergen challenge – lung function  (Group G, N=3) |  |  |  | Not implemented  **Resource constraint**: the investigator works alone and can see the intervention information from both the cage cards and the IT systems during data submission (**technological & operational constraints**).    **Knowledge constraint:** an automated measurement was used. Did not know you should mask when working with automated systems.  **Belief in the value:** researchers did not see that masking would improve the results when it was an automated system. |  |
| Pharmacodynamic studies (PD) for target engagement  (Group F, N=2) |  |  |  | Intervention information was masked. Samples are labelled with animal ID and experimental group ID. |  |
| Bone marrow biomarker studies – flow cytometry  (Group F, N=2) |  |  |  | Samples labelled with animal ID and experimental group ID.  Associated documentation shows the intervention (**operational constraint**).  During image quantification: **practical constraint**:  there is a need to be able to identify the control group data to support the gating steps of the flow cytometry data. | **Practical constraint**: during data processing as there is a need to be able to identify the control group data to support the gating steps of the flow cytometry data.  Masking is not implemented in the downstream analysis, see blockers described in the cell above. |
| Allergen challenge – *ex vivo* flow cytometry measurements  (Group G, N=3) |  |  |  | Samples labelled with animal ID and experimental group ID.  Associated documentation shows the intervention (**operational constraint**).  During image quantification: **practical constraint**: there is a need to be able to identify the control group data to support the gating steps of the flow cytometry data. | **practical constraint**: during data processing as there is a need to be able to identify the control group data to support the gating steps of the flow cytometry data.  Masking is not implemented in the downstream analysis, see blockers described in two cells above. |
| Histopathology  (Group H, N=1) |  |  |  | Samples labelled with intervention information. Inductive reasoning requires knowledge of group membership. To mitigate against this a second masked review step where intervention information is hidden, or peer review is implemented. | Not required: results are descriptive. |
