# Supplementary material for: A qualitative study of the barriers to using blinding in in vivo experiments and suggestions for improvement
Source: PLoS Biol. 2022 Nov 17;20(11):e3001873. doi: 10.1371/journal.pbio.3001873 (PMC9714947; doi:10.1371/journal.pbio.3001873)
Supplement: S3 Table — This table presents the data collected during the interview, including the randomisation and masking status for each step of the experiment, and the barriers identified for each study. In bold, we indicate the coding assigned to each barrier during our analysis. The coding assigned to a particular study are not fixed attributes of that study type but reflect the way these experiments were conducted. (DOCX) [file pbio.3001873.s003.docx]

**Supplementary Table 3:**

Results and analysis of the 12 interviews held at seven different UK universities in 2018-2019. This table presents the data collected during the interview, including the randomisation and masking status for each step of the experiment, and the barriers identified for each study. In bold we indicate the coding assigned to each barrier during our analysis. The coding assigned to a particular study are not fixed attributes of that study type but reflect the way these experiments were conducted.

| **Study and interview set up** | **How is randomisation implemented during the study?** | **How is masking implemented during allocation and intervention?** | **How is masking implemented during the conduct of the experiment?** | **How is masking implemented during the outcome assessment?** | **How is masking implemented during data analysis?** |
| --- | --- | --- | --- | --- | --- |
| Impact of chemogenetic receptor activation on behaviour  Species: mouse  Interviewer 1  N=1 participant | Randomised within block (cage): mice were allocated to receive either genetic material for the chemogenetic receptor or the control construct within each cage.  Order of administration of the active or control compound was similarly randomised and delivered in a cross-over design. | Not implemented  **Resource constraint:** single researcher prepares the intervention, completes the allocation and conducts the experiments which involves a full day commitment from the person performing the timed injections.  **Practical constraint:** visual differences between the compounds injected. | Cage cards include a code for each animal which did not refer to experimental group or intervention. | Cell counting: The sample has an ID that gives no information on the intervention nor experimental group.  Behaviour data: Screen set up without knowledge of the intervention nor experimental group. Data collected automatically via software, with threshold to define behaviour of interest set before the experiment. | Independent Analyst  & Randomly code the groups: analysis performed by student who only knows the experimental grouping but interventions were coded. |
| Neuronal recording under terminal anaesthesia  Species: rat  Interviewer 1  N=1 participant | Not implemented  Dose-escalation study, majority of animals received all treatments tested sequentially.  A small group of control animals received multiple vehicle injections. | Not implemented  **Resource constraint:** single researcher who prepared the drugs to be used on each day and performed the recording. This researcher also monitored the depth of anaesthesia and physiological parameters.  **Fear of errors:** the researcher did not trust students or other lab members to prepare the drug solutions accurately.  **Practical constraint:** drugs have to be administered in increasing dose order with vehicle first as the drug does not wash out. | Not applicable  This is a terminal study and allocation to experimental group occurs immediately prior to data collection. | Not implemented  The data is collected at the point the injections are administered by the single researcher who had prepared the injections.  Neurons continuously recorded via software, with markers set manually to indicate timing of injection.  **Knowledge constraint:** belief that automated measurements negate the need for masking. | Not implemented  **Resource constraint:** analysis performed by the single researcher. |
| Effect of diet in a model of heart injury  Species: rat  Interviewer 1  N=1 participant | Minimisation randomisation**:** rats assigned to experimental groups to balance average infarct size. | Not implemented  **Practical constraint:** visual differences between the diets. Cages for different diets were segregated on the rack to avoid cross-contamination. | Not implemented  **Practical constraint:** visual differences between the diets.  **Welfare concerns**: some of the diets worsen health outcomes and these animals needed to be easily identifiable by animal care staff.  **Operational constraint:** The cage cards show the intervention and experimental group. | Blood and imaging measurements performed by students unaware of intervention and experimental group. Animals are only referred to by randomised ID number.  **Operational constraint:** diet and cage cards reveal intervention and are visible during imaging capture and blood sampling. | Independent Analyst  & Randomly code the groups  Analysis performed by student who only knows the experimental grouping but interventions were coded. |
| Diabetes study  Species: mouse and rat  Interviewer 2  N=2 participants | Not implemented  Researcher relied on animal facility to allocate animals to diabetic vs control groups and had not realised this was done haphazardly not randomly.  Randomisation to pharmacological intervention was implemented. researchers used slips of paper in a bag to determine which pharmacological intervention each animal receives within diabetes/control groups. | Not implemented  **Resource constraint:** no other staff available to prepare the drugs that need to be prepared immediately before injection.  **Knowledge constraint:** had not considered masking but will code drug vials in future studies. | Not implemented  **Practical constraint:** diabetic animals visibly different to controls.  **Knowledge constraint:** researcher had not considered that the experiment could be masked to treatment within the diabetes/control groups. Will implement this in future. | The images were labelled with the animal ID. As a high throughput environment it was felt this was masked to intervention and experimental group (**knowledge constraint**). As the same researcher processed the images as conducted the earlier experiment there was a small risk of knowledge carrying through unmasking the study. In future, a new masked code will be issued for imaging.  Histology: slides analysed by a third party unaware of the intervention animals have received. | Not implemented  **Knowledge constraint:** had not considered masking during the data analysis. |
| Pathogen infection study  Species: mouse  Interviewer 2  N=2 participants | Not implemented  Researcher assumed animal facility weaned animals into groups randomly whereas in reality they were weaned as littermates. Will use independent system in future to ensure randomised experimental groups. | Not implemented  **Fear of error**: fear of making a mistake and giving antibiotic to the wrong animals | Not implemented  **Welfare concern:** animals receiving the pathogen without antibiotic receive more welfare checks | Not implemented  **Knowledge constraint:** had not considered masking during tissue sampling and microbiology measurement | Not implemented  **Knowledge constraint:** had not considered masking during the data analysis. |
| Effect of drug treatments on a surgical pain model  Species: mouse  Interviewer 2  N=1 participant | Not implemented  Researcher had not appreciated that haphazardly allocating animals to experimental groups was not random. Will use an independent system in future. | Surgery: assistant reveals to the surgeon whether the animal was undergoing a sham or full surgery only once the ligament to be cut was exposed.  Drug treatment: Assistant coded the animals and the drug vials.  **Practical constraint:** the researchers weigh the animals to calculate the dose before injecting the drug and knew which animals were grouped together but not the intervention they received. | Researcher and animal care staff masked as cage cards carry coded information hiding both the intervention and experimental group details. However, welfare assessments will indicate which animals are part of the model induced groups (**practical constraint**).  Assistant will reveal code if the animal care staff need to know what treatment an animal has received. | Surgery: animals were coded but the sham group gradually becomes apparent during weekly behaviour testing.  Drug intervention: assistant had re-coded the animals following drug injection to ensure that the researcher did not know which intervention they have received.  Histology: slides were coded and scored by two researchers independently. | Randomly coded the groups  Researcher analysing the data knew the experimental group but interventions were coded. |
| Embryonic tissue transfer  Species: mouse  Interviewer 2  N=1 participant | Not applicable    Genotype of embryos unknown at point of transfer into hosts. | Embryo genotype: researcher was masked because there was no visible difference between the embryos. Dissection and implantation into the host were conducted before the genotyping results return.  Host genotype:  Not implemented  **Resource constraint**: masking would require an additional person. | Not implemented  **Knowledge constraint:** had not considered masking the host or the tissue genotypes. | Not implemented  **Operational constraint:** the hosts’ genotypes are visible on the request system from the animal facility.  **Knowledge constraint:** had not considered masking the genotype of the tissue implanted.  **Resource constraint:** masking would require the help of another person and other lab members are too busy.  **Fear of errors:** the researcher would be happy to mask aspects of the experiments that could be unmasked on the same day but would not risk not knowing the experimental group allocation for longer as a mistake would compromise the entire experiment. | Not implemented  **Knowledge constraint:** had not considered masking during the analysis. |
| Disease model development  Species: mouse  Interviewer 3  N=1 participant | Mendelian inheritance randomly allocates animals to experimental group (genotype).  Mechanical intervention to induce osteoarthritis:  Not implemented  Allocation not randomised. All animals from a single cage received the same intervention and the experimenter did not want to rehouse into treatment cages to avoid disrupting social ranking within cages. Multiple cages were used per experimental group. | Not implemented  **Operational constraint:**  cages labelled with genotype but not subsequent intervention.  **Practical constraint:** as different experimental groups have different phenotypes this unmasks the study to both intervention and experimental group. Therefore, there is no point to implementing blinding. | Not implemented  **Welfare concern & Operational constraint:** researchers were responsible for welfare checks and need to know the intervention received and genotype of the animals.  **Resource constraint:** masking would require the help of another person. | Blind to intervention and experimental group as the samples for tissue processing and histology identified by non-informative animal ID. | Not implemented  **Knowledge constraint:** had not considered masking during the analysis. |
| Circadian variation study  Species: mouse  Interviewer 3  N=1 participant | Not implemented  Allocation to experimental groups was based on the animal’s weight and condition, with those in poorer condition allocated to the control group. | Not implemented  Intervention and experimental group recorded on cage card. Animals in some groups are shaved giving a visual difference between animals. In future will shave all animals.  **Knowledge constraint:** unaware of the importance of masking. | Not implemented  Intervention and experimental group recorded on cage card.  **Practical constraints:** visible difference between the animals in different experimental groups. | Not implemented on sample collection.  **Practical constraint:** Timing of a cull was part of the intervention and this could not be blinded at the point of sample collection.  Implemented during sample processing.  Masked to both intervention and experimental group as histology samples were coded. | Not implemented  **Knowledge constraint:** had not considered masking during the analysis. |
| Neurovascular phenotypes in larvae.  Species: zebrafish  Interviewer 3  N=1 participant | Mendelian inheritance randomly allocates animals to experimental group (genotype). | Not applicable  Experimental group allocation depended on the genotype which led to development of a spontaneous trait; thus, researchers were inherently masked as they could not predict which animal would have which genotype and develop the trait. | Not implemented  **Practical constraints:** visible difference between the larvae in different groups.  **Fear of errors:** larvae in different experimental groups were placed in different 24 well plates due to a desire to reduce the risk of human error. | Not implemented  **Fear of errors:** larvae in the same experimental groups were placed on the same 24 well plates to reduce the risk of human error. The plates were labelled with intervention unmasking to intervention and experimental group. | Not implemented  **Knowledge constraints:** had not considered masking during the analysis. |
| Development of a tumour growth model.  Species: rabbit  Interviewer 3  N=2 participants | Randomised within block: with a pen blocking factor, animal ID randomly allocated to experimental group. | Cells to be inoculated were prepared and coded by the *in vitro* team masking the intervention (cell type and concentration) but not the experimental group for the *in vivo* team.  **Knowledge constraint:** were unaware of the need to mask to experimental group. | The animals were coded such the intervention was masked but the experimental group was identified.    **Knowledge constraint:**  were unaware of the need to mask to experimental group. | Masked to the intervention but not the experimental group as the measurements were conducted by the *in vivo* team.    **Knowledge constraint:**  were unaware of the need to mask to experimental group. | Randomly coded the groups  Researcher analysing the data knew the experimental group but not the intervention received. |
| Phenotyping and gene function study.  Species: zebrafish  Interviewer 3  N= 1 participants | Mendelian inheritance randomly allocates animals to experimental group (genotype). | Not applicable  Experimental group allocation depended on the genotype. Embryos from heterozygote crosses were processed in their clutch, thus researchers are inherently fully masked until genotyping was done at the end of the experiment. | Researchers were not aware of the genotype until genotyping was completed at the end of the experiment.  **Practical constraint:** visible difference between different genotypes. | Masked to the intervention and experimental group as the researchers were not aware of the genotype until genotyping was completed at the end of the experiment.  **Practical constraint:** visible difference between different genotypes. | Not implemented  **Knowledge constraint:** had not considered masking during the analysis. |
