# Supplementary material for: A qualitative study of the barriers to using blinding in in vivo experiments and suggestions for improvement
Source: PLoS Biol. 2022 Nov 17;20(11):e3001873. doi: 10.1371/journal.pbio.3001873 (PMC9714947; doi:10.1371/journal.pbio.3001873)
Supplement: Attachment — Submitted filename: Reviewer_response.docx [file pbio.3001873.s005.docx]

Dear Roland,

Thank you for your letter regarding our article “The reality of blinding during in vivo research: an exploration of engagement, barriers, and strategies to move forward.”

We were pleased with the positive reactions to the revisions and below provide our response to the new feedback . We look forward to hearing from you regarding this reworked manuscript.

Reviewer 1 feedback:
The revised version of the manuscript has a much more complete description of the study's methodology. There are a few points where I think further clarification would be helpful.

1. Even though the study was not designed to unpick whether career stage/status or other demographic variables impacts attitudes towards blinding, it would still be helpful to both report these and to have at least some commentary on which variables seemed to track with differences in the barriers identified - e.g., did industry researchers/senior researchers/staff members seem to be more attuned to some kinds of constraints than others? Likewise, it is still useful to report on which themes came up a lot and which came up rarely even if prevalence can't be estimated with more precision. One of the ways that an exploratory study like this one can add value is by helping future researchers get a sense of what the relevant sources of variation are in this area so that they can make a more educated guess about what factors they should control for in their study design, and as written at present the manuscript does not give a lot of information of that kind.

We appreciate that variables such as career stage could track with differences in the barriers identified. However, the study was not designed to explore this aspect. In both settings, for some experiments, multiple participants were interviewed together and the experiment discussed collaboratively. The data collected is therefore associated with the experiment not the participant. Regarding a comparison between industry and academia, with interviews being conducted from one industrial partner we felt the sampling breadth was not sufficient to enable such as comparison. These limitations have been added to the limitation section of the manuscript as follows: “For most experiments, multiple participants were interviewed together and the experiments discussed collaboratively. The data collected are therefore associated with the experiment, not the participant, and cannot be used to explore the effect of career level on attitudes and perceived blockers. As data were collected from only one industrial setting, this also prevents comparisons between industry and academic settings.”

2. The claim in the limitations section that the sample size should be sufficient to identify the majority of the barriers researchers might encounter doesn't seem justified to me. In the study cited in support of this claim (Faulkner et al 2003), the original authors are looking at how many people are needed to find problems in a single application (an employee timesheet), so all of the variation is in the positionality of the users. In the present study, differently positioned users are talking about different experiments, so there's variation on both ends.

To address this point, we have added the following sentences to the limitation section of the manuscript: “This recommended sample size is based on a single application, therefore our study design makes the assumptions that the barriers are equivalent across *in vivo* studies and not heavily dependent on experimental type. Data saturation was not assessed, which means that additional barriers might exist that our study did not detect.”

3. Reporting on data saturation could help with the problem identified above in (2): since the development of the coding scheme was iterative, the authors could report on how many interviews were needed to develop a reasonably stable coding scheme (that is, to reach saturation). If the authors reached saturation using only a subset of the articles, then I'd have more confidence in the claim that the majority of possible themes have been identified. For an example of this methodology, see Guest, Bunce, and Johnson ([https://doi.org/10.1177/1525822X05279903](https://protect-de.mimecast.com/s/aLlPCWPxl1HzJLEPGfyUaU6?domain=gbr01.safelinks.protection.outlook.com)). This article has also been very widely cited as evidence that ~12 interviews per group is generally enough to reach saturation, although what counts as a group (as discussed in 1 above) is a key question to be worked out!

As described in the methodology, the data was collected before we conducted an inductive iterative discussion of the data to develop the coding. Consequently, we have not assessed for data saturation and this is a limitation of the study design. We have added this to the limitation section of the manuscript as follows: “Data saturation was not assessed, which means that additional barriers might exist that our study did not detect.”

4. One of the other reviewers notes in their original report that it's ironic that a study on masking such as this one cannot itself be masked, but masking during data analysis is certainly possible. I'm not sure it would make a huge difference for this particular study (or how possible it would be given the pre-existing relationships between interviewers and interviewees), but given that this is a manuscript on masking it would probably be good to be precise about this in the methods section. Right now the methods section states that the data was "stripped of any identifying information," and it's not clear if this was simply de-identification of the type that the IRB would require to conceal participants' identities from members outside the study team, or if the authors were attempting to mask themselves so that they would be unaware which interview summary they were dealing with when developing and applying the coding scheme.

We have added the following information to the methodology on page 5 to provide further clarification of the masking during analysis:

“The removal of identifying information fully masked EJP and NPdS to the source of the data. Whereas NAK, CB and EJS were partially masked as they could recognise the data from the interviews they conducted themselves. “

In terms of the analysis, I have a few points.

1. First, one minor point: The sentence introducing the paragraph discussing the case of transient piloerection is unclear, in that I wasn't sure what review the authors were referring to until I went back and read the other reviewers' comments in more detail. It's a very useful example to include, but it should be clear that it's an example coming from a reviewer and not from a review of the interview data.

The text has been reworded to clarify that this section of the discussion is based on concerns raised by the scientists during the interviews: “A number of interviews raised concerns around managing welfare needs whilst maintaining masking. The scientists raised examples where some interventions or model induction have a transient welfare issue that would be tolerated for certain experimental groups. For example, in oncology studies, the anti-cancer treatments being tested could induce moderate transient piloerection or hunched posture and reduced social interaction which would be tolerated for a short period of time in the treated animals with additional monitoring but the same welfare concern would not be tolerated in the control animals.”

2. And now, the major point: Throughout the results section, it is difficult to tell whether the authors are reporting on what interviewees told them or making recommendations about what best practice is. For example, in the very first paragraph of the results section, the manuscript states that masking was not relevant for a number of the experiment types included—is this the authors' determination, or the interviewees' assessment? Likewise, did participants agree that in 28/30 cases that masking could be implemented in some stages to reduce bias? Did participants rank different masking strategies as low/moderate/high quality? Did participants express concern that power dynamics or a want to please might decrease the effectiveness of bias reduction strategies? Some rewriting is needed here so that readers can easily identify which elements are opinions held by the participants and which ones are judgements/recommendations from the authors.

We have added a new heading: Discussion, to clarify where the results section ends and where our discussion/recommendations start. The discussion section covers pages 10 to 26. We also made the following amendments to address the specific points the reviewer raised:

Example 1: *For example, in the very first paragraph of the results section, the manuscript states that masking was not relevant for a number of the experiment types included—is this the authors' determination, or the interviewees' assessment?*

On page 10, the title of table 2 was changed to:

“Experimental scenarios identified during the interviews for which masking was not applicable (as assessed by the authors).”

Example 2: *Likewise, did participants agree that in 28/30 cases that masking could be implemented in some stages to reduce bias?*

On page 7, in the first paragraph of the results we have rephrase the sentence to:

“As part of our analysis, we assessed the experiments to consider when or whether masking was relevant; for six of the experiments discussed, masking was not required at one or more stages of the experiment due to the experimental design implemented (Table 2).”

Example 3: *Did participants rank different masking strategies as low/moderate/high quality?*

Table 3 (page 12) and table 5 (page 23) now sit in the discussion section, which clarifies that these arise from the evaluation of the interview results. We also added the following legends to both tables: “We have classified the masking options as either low, moderate or high based on the ability to minimise risk of bias.”

The title of Table 5 was changed to: “Example of strategies to implement masking during data analysis” to clarify that these strategies had not all be identified during the interviews. We have also added to the table heading Strengths and weakness “identified by the authors or the literature”

Example 4: *Did participants express concern that power dynamics or a want to please might decrease the effectiveness of bias reduction strategies?*

On page 20, in the discussion section, we have rephrased the sentence to “Power dynamics, or a want to please (whether knowingly or otherwise) could also be at play, particularly when the support staff is junior and part of the same department, decreasing the effectiveness of this strategy to reduce bias.”

We also reviewed the manuscript; the following amendments were also made to be more explicit:

On page 12, we have changed the word review to interviews

On page 20 we reworded the sentence regarding the IRWIN test to avoid confusion that it might be related to the reference rather than the study captured in supplementary table 2.

On page 21, we have changed the word review to interviews to be more explicit in the sentence “Our interviews found that samples were often labelled with both a masked sample ID and a group ID.”

On page 21, we have changed the word initial review to interview to be more explicit in the sentence “This was seen for the flow cytometry studies where the interview found ...”

On page 25, we have rephrased “during discussions with scientists” to “during the interviews”.

On page 26, we have reprhased “During the review process” to “While analysing interview data, we”

On page 27, we have added “within a facility” to “This suggest that an internal review within a facility, ...”

3. If it is the case that many of these assessments about the quality of various strategies etc are actually the authors' judgements and not reports of participants' assessments, then I think the authors should be much more upfront about the fact that they are offering their opinions on what counts as a high/low quality strategy, rather than offering guidelines of the type that would be more typically developed through a broad consultative process. This seems especially important given that many of the authors have been involved in the production of the ARRIVE guidelines and so readers might be more inclined to read what's written here as a guideline rather than as an exploratory study that provides a starting point for future discussion (which I think is what the authors intend).

As addressed in point 2 above, we have endeavoured to be more explicit where it is the author’s judgement. To address the reviewers concerns, we have also added to the limitation the following sentence “The recommendations presented in this paper have not been produced via a broad consultative process, they are based on the interview findings and subsequent analysis by the authors.”

Reviewer #3:

The authors have made a considerable effort to reply to the reviewers' comments, and were able to address most of them in a meaningful way. I have divergences of opinion with them, but this should not disqualify an academic paper from being published, in my view.

I have two final comments, that I will leave to the authors' - or editor's - judgement whether to consider or not.

1. The authors decided to accommodate the other reviewer's suggestion to change "blinding" to "masking", even though it is not standard terminology (e.g. one does not usually refer to "double masking"). I'm not surprised to see this sort of request, in this day and age, and though certainaly well-intentioned, I find it utter nonsense. Perhaps this is because English is not my mother tongue. So I do find it odd that representatives of an organisation that does not use this terminology (vide the ARRIVE guidelines themselves) would be so quick to change it upon request, despite adding to the already confusing semantic chaos, not being the term used in the interviews, or registered in the notes taken, or originally used in the manuscript (as the wording now used in the sentence justifying this choice seems to suggest, which I couldn't help connect with the practice of HARKing). I find it nonsensical but if the authors can live with that decision, who am I to disagree?

We acknowledge that to date the word masking hasn’t been used as frequently but understand the perspective raised and agree with Reviewer 1.  The terminology around blinding is not without challenges, the reviewer mentions the term “double blinding” as an example; this term is ambiguous in the context of clinical research, and meaningless in the context of animal research. The term masking has been used previously in the context of *in vivo* research, for example the seminal paper published in 2008 by Prof M Macleod ([https://journals.sagepub.com/doi/10.1038/jcbfm.2008.101](https://protect-de.mimecast.com/s/cUpMCXQy0PuBwPQq0uWozQ9?domain=journals.sagepub.com)). Changing terminology is always challenging. We have added the term masking to the glossary.

2. Changing p-value to "statistical significance" does little to make sense of this claim: "This can then lead to animals being handled or data collected more consistently within each experimental group which may introduce bias and could potentially increase the statistical significance of a difference between experimental groups." It is not the "statistical significance" we are affecting, but rather the potential direction or magnitude of differences in the data collected by changing how animals "perform" (hence 'performance bias'). A p-value is a conditional probability of the observed differences in the data being as extreme or more if there were no effect, and it is affected not only by differences between groups, but also the variability in the data, and the statistical power. I understand what the authors are trying to say, but referring to 'statistical significance', here, is imprecise. However, wrong as it may be, it is not a hill I wish to die on, as I would not
want to revise this manuscript yet again, so I will leave it to the editor's judgment.

The reviewer raises a good point and the sentence on page 10 has been corrected to “This can then lead to animals being handled or data collected more consistently within each experimental group which may introduce bias leading to misleading conclusions (either leading to apparent differences where in reality there are none, or by masking real differences).”
