# Supplementary material for: A qualitative study of the barriers to using blinding in in vivo experiments and suggestions for improvement
Source: PLoS Biol. 2022 Nov 17;20(11):e3001873. doi: 10.1371/journal.pbio.3001873 (PMC9714947; doi:10.1371/journal.pbio.3001873)
Supplement: Attachment — Submitted filename: Blinding Manuscript reviewer comments.docx [file pbio.3001873.s004.docx]

Dear Roland,

Thank you for your letter regarding our article “The reality of blinding during in vivo research: an exploration of engagement, barriers, and strategies to move forward.”

We were pleased with the positive reactions of the reviewers and have found their feedback very helpful to refine the article further. We provide a much-revised version that we believe addresses the reviewers’ comments as detailed below. We look forward to hearing from you regarding this reworked manuscript.

**Reviewer #1:**

This manuscript reports on an interview-based qualitative study of researchers' self-reported practices and perceptions of blinding/masking in preclinical biomedical research. The data presented in this study is quite unique since there are at present very few qualitative studies of research practice and reporting behaviors, and therefore in my opinion quite valuable. But, the reporting (in particular the reporting of the methodology) is very incomplete, which makes it difficult to judge the quality of the interpretive work being done here.

It is pleasing to see that the reviewer finds the work unique and valuable. We appreciate the feedback on the methodology and as detailed below we have extensive rewritten the methodology to address these concerns.

1. The authors should include a qualitative research reporting checklist with their manuscript—either SRQR or COREQ would be appropriate, but I think COREQ would be the better choice here since this is an interview-based study. Many of the problems noted below would be addressed by working through this checklist.

We have worked through the SRQR checklist and added any missing information into the manuscript. We chose SRQR over COREQ as it is more recent and included COREQ as part of its development. We have submitted the checklist as part of the reviewer response.

The title was consequently changed to: “The reality of masking (blinding) during in vivo research: a qualitative study exploring engagement, barriers, and strategies to move forward”.

The following sentences were consequently changed in the abstract:

“The objective was to explore the awareness, engagement, perceptions, and the barriers to implementing masking in animal experiments.”

“*We conducted multiple interviews, to explore 30 different experiments, and found examples of excellent practice but also areas where masking was rarely implemented*.”

In the methodology section:

The following sentence was added : “*The goal was to understand how masking was implemented in practice for different types of studies at each step of the experiment and what the challenges and barriers to masking were.*”

Additional changes were also made to provide clarity over the methodology taken and are described in detail in response to comment 2 a-g.

2. The reporting of the methodology is very incomplete. The following elements at minimum should be included:

a. The sampling strategy used to identify participants. The manuscript currently states that this sampling strategy is nonrandom, which is fine, but the specific type of nonrandom sampling strategy needs to be stated (e.g. is it just a convenience sample, or is it a purposive sample, snowball sample, etc.). It would also be helpful to include as much information as is known about the population that declined to participate and how their demographic characteristics differ from participants.

The following has been added/amended in the methodology section to address this: “

“*In both types of settings we use purposive sampling. We chose a broad cross section of research settings to account for the possibility of cultural differences and to ensure we had identified the various barriers that could arise but the study was not designed to compare results between settings*.”

“*In the academic setting, NC3Rs Regional Programme Managers (ES, CB and KAA) interviewed researchers at seven different universities in the UK. We selected one or two research groups per university/facility to cover a variety of research species and research types. Different researchers were interviewed for each of the 12 experiments; all researchers approached agreed to participate but one interview was never scheduled due to the interviewer leaving the organisation (KAA).”*

“*In the pharmaceutical setting, with the approval of AstraZeneca Council for Science & Animal Welfare (Enterprise-wide governance group for research involving animals), a statistician embedded with the community (NAK) reviewed all study types conducted at the time at two distinct facilities in Cambridge, UK. This led to an assessment of 18 study types, which was a complete coverage of experiment type conducted internally within the UK. The project was advertised through the local ethical review board. Scientists who conducted these studies volunteered and were assembled into representative groups to be interviewed. The group size and which studies they were interviewed about is captured in Supplementary Table 2*. “

b. The number of interviewees and their demographic characteristics. Right now we only know how many types of studies the interviewees described, but we don't know if one interviewee described multiple studies; if the same study types were described by multiple interviewees; if the same study types were described by participants in both academic and pharmaceutical settings, etc. The lack of this information makes it extremely difficult to differentiate between concerns that are due to the type of study, the knowledge/perceptions of the researcher, and the institutional setting.

To address this, for the academic setting the following has been added to the methodology: “*In the academic setting, each experiment was described by a different interviewee*.”

To address this for the commercial settings the following has been added to supplementary Table 2: “*There were 8 interview groups where the number of researchers in each group varied from 1 to 3. No researcher sat on multiple groups. Which group discussed which studies are captured in the study type column.”*

There is no overlap in experiment type (in terms of objective of the experiment) between the commercial and academic settings. There is overlap in terms of species. Our study was not designed to attempt to differentiate whether these issues were due to a subset of experiments or situations.

We have added the number of interviewees and clarified that they were the scientists conducting the experiments themselves. We have not added further demographic information due to concerns that this would risk identifying the participants.

To increase clarity and address these questions, we have added a section titled ‘Limitations of the methodology’ and it includes the following:

“*Limitations of the methodology: The interviews were conducted by different individuals across the dataset and different interviewers might have asked different questions depending on their background knowledge. The sample is not random, rather the study was designed to encompass a broad range of experiment types. The sample size is small but should be sufficient to identify the majority of the barriers researchers can come across [25]. This study aimed to identify the themes around barriers to using masking in preclinical research, but not to quantify them or test a specific theory. As such, the coding for the themes identified in the interviews was developed as the coding took place (inductive coding). Although this approach is appropriate to identify frequent themes where no similar research has been conducted previously [26], it does have some limitations: there is risk of confirmation bias, and quantitative analysis cannot be performed on the resulting data. As this study has identified common themes and developed some coding, it can be used as a basis for future studies to develop a code set a priori and use it to test specific theories about masking in preclinical research*.”

The design of the survey and the ethical approval that was granted did not allow us to capture the demographics of the interviewees. We have added to the methodology the following to clarify this and provide an explanation why:

“*This format was designed to encourage open and honest reflection; both animal research and questionable research practices are sensitive topics which can hinder people sharing. For that reason, the interviews were not recorded and interviewers were explicit that any details allowing the identification of the research group would remain confidential*.”

c. Whether an interview guide was used, and if the interviews were structured/semi-structured/unstructured. Right now we only know that interviews focused on blinding at four different stages of the research process, but there is no other information on steps the research team took to ensure that the same types of questions were asked across sites; whether there are important considerations about question order that were taken into account in the interview guide, etc. Information should be reported on which study team member interviewed which participant, and how the background knowledge of the study team member might have impacted the questions asked. This is particularly important if an interview guide was not used.

We have added to the methodology to provide clarity of the interview structure and the following sections address these questions:

“*All interviewers (NAK, ES, CB and KAA) had lab experience, a PhD and formal scientific training, and between 7 and 14 years’ experience working as, or supporting, in vivo researchers. In both settings, the interviewer and the researchers had an existing work relationship. We were aware that masking was not widely used in animal experiments but believed that the research community was open to using it and assumed there were practical barriers stopping them. The interviews were semi-structured (see Supplementary Table 1) and conducted as a discussion with the question order not considered. This format was designed to encourage open and honest reflection; both animal research and questionable research practices are sensitive topics which can hinder people sharing. For that reason, the interviews were not recorded and interviewers were explicit that any details allowing the identification of the research group would remain confidential. The agreement with the researchers did not include consent to share quotes nor detailed transcripts of the interview.*

*The brief to the interviewers was to explore how the experiments were conducted, assess whether, how and when masking was used to conceal the intervention the test subject had received, or the experimental group they belonged to, from the researchers running the experiment. When masking had not been used, the interview also explored the reasons for not using it and whether it would be feasible to implement in future experiments*. “

We have added Supplementary Table 1 with the details of the interview guide

We have added to Supplementary table 2 which interviewer was involved in which interviews. We have clarified in the methodology that NK was the interviewer for the experiments in the commercial setting.

We have also extended the study limitations section to include:

*“*The interviews were conducted by different individuals across the dataset and different interviewers might have asked different questions depending on their background knowledge*.“*

d. Who conducted the qualitative data analysis (QDA), and what analytical approach was used. The interpretive approach appears to be more of a content analysis/thematic coding rather than a grounded theory analysis based on the information provided in Table 5. In addition, we need to know whether this approach was inductive or followed a pre-specified code book, whether an iterative approach was used, whether constant comparison was employed, etc.

The following additional information has been added to the methodology

“Following the interviews, the data were discussed as a team (all authors excluding JCU) in an inductive iterative process to identify the themes and develop a coding strategy to classify the responses (Table 1). For each experiment, the coding assigned along with the relevant data for this assignment is presented in Supplementary Table 2 and 3.*“*

We reviewed the data provided and ensured that the text clearly distinguished the descriptions and the assigned codes. We also ensured that terms were consistently used and removed the application of the codes from the randomisation step of the experiments.

e. What measures and thresholds of intra-rater reliability were used, and if multiple study team members were involved in the QDA process, what measures of inter-rater reliability were used. Were there any thematic codes on which the research team failed to achieve an acceptable level of IRR, and if so were these dropped from the study? A full description of the codes/coding tree should also be provided.

As classification was achieved by consensus post-interview, an IRR measure is not applicable. We have added Supplementary Table 1 with a definition of all codes used.

f. What measures of data saturation were used. In particular, the study should report whether termination of data collection was determined by interviewee characteristics or by saturation in the QDA process.

Data collected in the academic setting was determined by a sampling plan to provide variety of experiment type, species and settings. Data collected from the commercial setting was a complete coverage of experiment type conducted internally within the UK. This has been added to the methodology.

g. Major themes and minor themes should be reported so that the reader can get some sense of how prevalent different concerns about blinding were, and whether the prevalence of concerns relates to career stage, biomedical research field, academic vs. industry location, local cultures around animal welfare, etc. Positions not taken in the data should also be reported, and analysis of positions not taken should be considered as it can be very fruitful in cases such as this one where there are potentially sensitive topics that may arise (e.g. questionable research practices that could be interpreted as misconduct, animal welfare concerns, power dynamics between junior and senior lab members). See Adele Clarke's _Situational Analysis_ for an excellent description of how to analyze positions not taken.

As this was an explorative research to understand the blockers to masking that were common across studies and institutes, we did not design the study to explore whether the prevalence of concerns relates to factors such as career stage. This is beyond the scope of what we were trying to do. We have added to the methodology to give greater clarity with

“*In both types of settings we use purposive sampling. We chose a broad cross section of research settings to account for the possibility of cultural differences and to ensure we had identified the various barriers that could arise but the study was not designed to compare results between settings.”* We also amended the abstract from stating it was a qualitative review to *“we conducted exploratory interviews*”.

Furthermore, we have added to the conclusions the limitation of these study and the potential for further research “*This research provides a platform for further qualitative research to explore these issues in more depth, for example explore whether the prevalence of specific barriers relates to the career stage, environmental setting (e.g. academic versus industry), or biomedical research field*.*.”*

Regarding positions not taken, as described now in the methodology, this was an explorative assessment of blinding and the codlings were developed in response to the information obtained. The data collection process was designed to minimise the risk of omission due to the sensitivity of the topics. We have added the following as a limitation of the methodology: “*This format was designed to encourage open and honest reflection; both animal research and questionable research practices are sensitive topics which can hinder people sharing*.”

3. The manuscript at present contains no representative quotes from the actual interviews. This is quite unusual for a qualitative study and means that the reader has not been provided any opportunities to compare the original data against the interpretations made by the study teams. For example, in Table 5, rather than paraphrasing participants' concerns, the table should put representative quotes next to their respective coding classifications. Ideally the demographic characteristics of the interviewees should be reported along with both quotations and paraphrased findings throughout, for the reasons described in 2b above.

To encourage researchers to speak freely, we did not capture a full transcript of the interview and during the recruitment process we had been explicit with the researchers that we would only be sharing summary details. We felt this was critical to allow full honestly in the conversation as to what was actually happening during the experiments. In this community, sharing research goals and species can identify scientists.

We have added to the methodology to be explicit in the approach we have taken.

*“This format was designed to encourage open and honest reflection; both animal research and questionable research practices are sensitive topics which can hinder people sharing. For that reason, the interviews were not recorded and interviewers were explicit that any details allowing the identification of the research group would remain confidential. The agreement with the researchers did not include consent to share quotes nor detailed transcripts of the interview. “*

We have added to the limitations section that we have not captured quotes/raw data to allow assessment of our interpretations.

*“The construct of the study did not allow the capture of quotes or raw details and thus this raw level of data is not shared and limits the assessment of our interpretation.“*

We have also added to the conclusion that this explorative research provides a starting platform for more detailed research program to unpick these issues.

“*This research provides a platform for further qualitative research to explore these issues in more depth, for example explore whether the prevalence of specific barriers relates to the career stage, environmental setting (e.g. academic versus industry), or biomedical research field*”

4. When the authors say that the data are fully available without restriction, do they mean the full interview transcripts? If so, where are these made available?

The data we present is based on the interview notes, this is provided in Supplementary Tables 2 and 3. We have explained what we collected in the methodology:

*“Following the interviews, the data (study type, randomisation strategy, the masking level achieved and the associated barriers) were tabulated and stripped of any identifying information. These data were discussed as a team (all authors excluding JCU) in an inductive iterative process to identify the themes and develop a coding strategy to classify the responses (Table 1). For each experiment, the coding assigned along with the data on which this assignment is based are presented in Supplementary Table 2 and 3.”*

We also explained why we do not have quotes or transcripts from the interviews:

*“The interviews were semi-structured (see Supplementary Table 1) and conducted as a discussion with the question order not considered. This format was designed to encourage open and honest reflection; both animal research and questionable research practices are sensitive topics which can hinder people sharing. For that reason, the interviews were not recorded and interviewers were explicit that any details allowing the identification of the research group would remain confidential. The agreement with the researchers did not include consent to share quotes nor detailed transcripts of the interview.”*

5. The authors might consider using "masking" throughout as an alternative to "blinding," given that the use of blinding in this context promotes an ableist stereotype that being blind can be equated with lacking knowledge.

We have added the following sentence to the introduction:

*“In this paper, we choose to refer to this process as ‘masking’, which has not been used as frequently as ‘blinding’ in the context of in vivo experiments but there has been a move away from the term ‘blinding’ as it associates being blind with lacking knowledge and perpetuates negative stereotypes about blind people”*

We have changed the terminology throughput the manuscript to the word masking.

We have used both terms in the title to ensure the community will understand the paper content.

6. I suspect another round of review will be needed here, because it is difficult to assess the quality of the interpretive work performed and the solutions proposed without the methodological detail outlined above. For example, I am inclined to agree with the authors' argument that masking by assigning tasks to less knowledgeable/invested lab members is an insufficient strategy, but it would be useful to know if the study included interviews with any technicians/grad students/junior lab members so that we know if there are discrepancies between PIs' perceptions of these employees' knowledge versus their employees' self-reported awareness of the expected outcomes. In other words, while I am inclined to agree with this argument based on my own prior experience, it's difficult to know at present whether this is an argument that is supported by the data collected or whether it's a hypothesis that should be investigated in future studies. Once the manuscript includes more detail about the participants included in the study, then the presentation of these arguments can be more easily assessed.

As now detailed in the methodology, we only interviewed those conducting the research as we did not design the study to unpick the perception as a function of seniority. This could be researched further and this point has been added to the conclusion “*This research provides a platform for further qualitative research to explore these issues in more depth, for example explore whether the prevalence of specific barriers relates to the career stage, environmental setting (e.g. academic versus industry), or biomedical research field.”* In the results section we therefore used terms such as “has the potential” or “power dynamics could be at play” to represent these are hypothetical potential risks.

7. Finally, one small note—one study that the authors should be aware of is Reichlin et al 2016 (10.1371/journal.pone.0165999), which addresses the question of whether the lack of reporting on blinding in the literature correlates to lack of blinding in practice. Contrary to what the authors argue in their introduction, Reichlin et al's data suggests that blinding, randomization, and sample size calculations are all performed more often in practice than they are reported in publications (although it is worth noting that their survey is based on self-reported data).

We have added a sentence in the introduction to include Reichlin work “. *A self-reporting survey of researchers in Switzerland found that 27% (sample size = 530) had implemented masking at outcome assessment, but the survey authors noted that the self-reported rates were likely biased as they were considerably higher than the reporting rates found by systematic review [23]*.” The self-reported data for blinding at allocation was only 27% which was in keeping with our message that blinding rates are still low.

Reviewer #2:

The manuscript by Karp et al is an important and timely contribution to improving the quality of in vivo research, addressing application of blinding in different stages of experiment. I think it is very well written, based on the experience of authors and backed up by interviews and case studies.

It is pleasing to see that the reviewer finds the article well written, timely and an important contribution.

1. As the principle applies across species and study settings, I would only suggest to make (re-phrase) the statements to reference 1 and 2 in the introduction more general. Especially the work of Rosentahl and Fode (1) is so classic example which goes well beyond rats - ie it does not matter if you are studying worms, rats or humans, the recordings are biased without proper blinding.
#broaden introduction to wider species

To make it clearer that this work is generally applicable, we have extended the section in the introduction to references multiple species and it now reads as follows:

*“For example, in rat studies, particular expectations regarding the animals’ performance in different experimental groups can lead the researchers to subconsciously handle the animals differently and create spurious results [2]. Researchers’ expectations can also influence the observations themselves. In a study of pig behaviour where the same video was altered slightly and shown to researchers twice but with different labels, researchers were shown to score identical behaviours differently, based on priming through the video label [3]. Similar effects have been replicated in studies with a variety of species (e.g. in cattle, chickens, ants and flat worms)[3-5]*.”

Reviewer #3:

This manuscript reports the results from a qualitative study focusing on scientists' knowledge and perceptions on blinding of experiments to improve their reliability and reproducibility.
Before I proceed with my comments, a declaration of interests: I'm familiar with the work of some of the authors, most of which I consider of high quality, which might also bias my appraisal. On the other hand, I have been critical - often publicly - of the organization for which some of them work in regard to the overreach given to the ARRIVE (granted, not always by said organisation itself) as a tool for the planning of experiments, for which these guidelines were not originally designed.
As regards the manuscript itself, I find it has merits, it is well written, and I appreciate the depth of analysis of the issues at stake. I do have, however, to make the following remarks:

It is pleasing to see that the reviewer finds the article well written and appreciates the depth of the analysis.

1. Although the research goal is laudable, the design has limitations, one of which being, ironically, that the study cannot be blinded at any stage, which is not acknowledged by the authors. And it should be, particularly given the topic covered.

As our study aims to identify common themes that hinder scientists from implementing masking through interviews, blinding is not an applicable strategy. However, the original draft lacked methodology detail which has now been addressed (see reviewer 1 question 2). Furthermore, we have also added a section called limitations of the study to reflect on this topic (see answers to reviewer 1 question 2b)

2. It is not clear from neither the Methodology section nor the Contributions section who carried out the interviews, when, and whether the interviewers were randomly allocated to the interviewees (if not, the possibility of bias should be acknowledged, even if no statistical analysis of results was performed).

The methodology section has been extensively reworked to provided better clarity on how the study was set up (see answers to reviewer 1 question 2). Furthermore, we have also added a section called limitations of the study to reflect on this topic (see answers to reviewer 1 question 2b)

3. Authors state that: "The quality of the strategies varied in the ability to minimise risk of bias being introduced and highlight that blinding is not simply a 'yes, it is blind' or 'no, it is not.' The strategies exist on a spectrum of effectiveness. Ideally blinding needs to not only conceal the intervention information (e.g. drug X) but also the experimental group membership (i.e. which animals are grouped together in group 1, group 2 etc) during allocation, conduct and outcome assessment"
While it is hard to disagree with this statement, it does however raise an issue in regard to the quality of information provided in systematic reviews trying to assess quality of research, in which blinding is often neither categorized into the categories described by the authors, nor described in a nuanced way, and rather reported as a "yes/no" dichotomy. I would indeed appreciate seeing this issue discussed, particularly considering the connection of the authors to the practice of carrying out systematic reviews.

We agree with the reviewer, that the reality that blinding is more nuanced than a binary yes or no is a potential issue for systematic reviews. The need to aggregate data in a meaningful way in systematic reviews warrants a pragmatic approach and a simplification of the reality. This isn’t just an issue for blinding but affects any other measures of good practice such as power analysis or ethical review. We however feel that this point is beyond the scope of this manuscript and would add to an already long article.

4. I am not convinced by the authors' statement that "[lack of blinding] can then lead to animals being handled or data collected more consistently within each experimental group which will reduce the variability seen and could potentially increase the statistical significance of a differences between experimental groups. The argument is presented in a quite diffuse and convoluted manner, and does not provide any references to back it up. It also, in my view, misconstrues what "statistical significance" means. There is moreover a typo ("of a differences"). I agree that the biases resulting from lack of allocation concealment and blinded outcome assessment can lead to an overestimation of effects, particularly because there is evidence in this regard, yet the argument that it reduces variability in such a way that analysis of results will yield smaller p-values, aside giving p-values more importance than they deserve, has not, to my knowledge, been established in the
literature.

Thank you to the reviewer for spotting this. It was poorly worded and should have focused on bias not variability. The sentence has been reworded to correct this. It now reads: “*This can then lead to animals being handled or data collected more consistently within each experimental group which may introduce bias and could potentially increase the statistical significance of a difference between experimental groups*.” The typo ("of a differences") has also been corrected.

5. The authors refer profusely to the ARRIVE guidelines on the matter of blinding of experiments, in a manuscript where the focus is how researchers plan and carry out their experiments, rather than their reporting. Either the authors explicitly make the case that reporting guidelines (theirs in particular or reporting guidelines in general) can be reversed-engineered to plan an experiment, or should be more cautious with such claims. No mention of guidelines on planning of experiments, such as the PREPARE, are made. The latter is particularly unfortunate, since the communication and planning warranted to orchestrate the combined efforts necessary to make multiple persons carry out different, blinded, tasks, is better addressed by the PREPARE. The authors should have realised this upon stating that "The recommended approach of a common welfare strategy is a significant shift and will require exploration of the topic with all the stakeholders to agree the welfare assessment
protocol going forward".

As the PREPARE guidelines do not mention blinding, we had not referenced them in the manuscript. However, as we have extended the section on discussion of welfare issue management we have now included a reference to PREPARE and the additional text is explicit about the planning needed to manage welfare and blinding.

*“In these situations, a harm-benefit analysis is needed to explore the ethical challenge of the risk of bias over the risk of harm for individual animals. Where it is found that unmasking will (or may) be required, a clear plan should be put in place in advance of the experiment starting so as to determine which individuals are to be unmasked, and at what stage this may be required. In discussing the plan, conflicts may be revealed between different parties, but it is important that decisions are reached so all parties know how to proceed in the event that a risk of bias is to be introduced; this will help to keep any bias to a minimum. The PREPARE guidelines can help with this as part of the suggested dialogue between the scientists and the animal facility [31].”*

6. The ethical issues arising from non-disclosure of treatments (in the broadest sense of the term "treatment", which could well mean "tumour implantation") to animal care staff is underexplored. Knowing the interventions informs animal care and clinical decisions, and the example given by authors of a rat showing piloerection and hunched posture being treated differently according to the expected outcome is a good example. It makes a world of difference in preventing unnecessary suffering that caretakers and vets know whether a clinical sign is a transient state or the first step towards irreversible decline in health and welfare, which may depend on the assigned treatment (or lack thereof). In the context of understaffed animal facilities, allocating manpower for more frequent monitoring of the animals that are more likely to quickly progress to severe stages indeed makes sense and allows for earlier humane endpoints, and thus for more humane science. The impact of
different professional objectives (carry out research vs. caring for animals, even between scientific vs. deontological duties) may lead to conflicts between scientists and animal care staff, which must be acknowledged, discussed, and sorted beforehand. The ARRIVE (and, to be fair, almost any other set of reporting guidelines) provide little to no guidance on how to accomplish this.

Whilst we had referred to the need to undertake a harm-benefit analysis to explore the balance between minimising risk of welfare concerns and introducing bias, we agree that this required more emphasis and so have extended the discussion. Discussion around conflicts has also been added, but with the focus being on needing a plan in place to ensure all parties are aware of who is to be unblinded and when, rather than further exploring the reasons behind the conflicts as these are beyond the focus of the manuscript. A reference to PREPARE has been added as this discussion would fall under the dialogue required between the scientists and the animal facility.

“*In these situations, a harm-benefit analysis is needed to explore the ethical challenge of the risk of bias over the risk of harm for individual animals. Where it is found that unmasking will (or may) be required, a clear plan should be put in place in advance of the experiment starting so as to determine which individuals are to be unmasked, and at what stage this may be required. In discussing the plan, conflicts may be revealed between different parties, but it is important that decisions are reached so all parties know how to proceed in the event that a risk of bias is to be introduced; this will help to keep any bias to a minimum. The PREPARE guidelines can help with this as part of the suggested dialogue between the scientists and the animal facility [31]. “*

7. Another underexplored issue is how abiding to the standards proposed in this manuscript is dependent on the available resources. I know cases where the planner of experiments, executer of said experiments, caretaker (including being responsible for cage change), data manager, and analyst are one and the same person. This might be an extreme scenario, but not that uncommon, even in Europe. This economical bias is even harder to tackle than the biases the authors aim to address, and it may exclude science - and scientists - from lower-income countries from publishing in journals with higher visibility, or at all. This is not a criticism of the authors' call for higher standards, but rather a statement of fact, which I suggest the authors reflect upon in their discussion.

We were explicit in the conclusions that cost can be a limiting factor to implementing blinding. Nowhere do we state that experiments should be excluded if they are not blinded. This suggests we need to be explicit on this topic. We have therefore added to the conclusions the following: “*Aiming for a perfect solution for all experiments is unattainable but if, as a community, we embrace the concept that masking is on a spectrum and value movement along that spectrum, we enable researchers to make small incremental changes that reduce the risk of bias. Experiments that are not masked are still valuable to publish but this information should be disclosed to enable readers to assess how this affects the reliability of the results*.”

8. The authors are right in acknowledging that fear of errors from blinding is not entirely ungrounded. It could be added that the chances of errors increase with the number of people involved and the number of blinding (and un-blinding) steps necessary.

As suggested we have added: “*The chances of errors increase with the number of people involved and number of masking/unmasking steps necessary.*“

9. I disagree with the authors' claim that support staff is not "emotionally invested" in the research outcome. Indeed, when research support staff are acknowledged to be an important integral part of the broader institutional research team and hence of the research success (and they should be), within the establishments where they see themselves as valuable and valued, they are indeed invested that experiments yield "positive/good/meaningful" results. To disregard them as either not caring or not understanding the research objectives is quite misguided and tone-deaf, and indeed incompatible with a culture of care. The authors are hence invited to reflect upon whether it is not more effective to refer to blinding of the support staff as a process where the latter are involved and the reasons for why they are blinded to treatment allocation and other features are openly discussed. If not for any other reason, because all humans are hardwired to find patterns and connections
(even non-existing ones) anyway, regardless of being "invested" or otherwise, which could lead to performance bias and skewed results.

We agree that the use of the words ‘emotionally invested’ was incorrect, and so have re-phrased this paragraph:

“*However, whilst the support staff may be less inclined towards a particular experimental outcome, they will likely be aware of the goals of the researcher which has the potential to introduce bias if the support staff are aware of the intervention received. Furthermore, power dynamics, or a want to please (whether knowingly or otherwise) could be at play, particularly when the support staff is junior and part of the same department, decreasing the effectiveness of this strategy to reduce bias.*“

10. The authors refer to the practice of allocation concealment as "blinding during the allocation and intervention". It seems as it is to follow the description used in the ARRIVE, where different measures carried out for different reasons are neatly gathered under the same category of "blinding", perhaps to fit into the Essential 10 (who does not love a round number?). I am aware that the authors acknowledge the term "allocation concealment" as synonymous to their wordy terminology, but using the same term "blinding" for several contexts may confuse some readers, especially those more familiarized with e.g. the Cochrane terminology. A very brief discussion (i.e. one sentence, perhaps in the abstract/introduction)) on how the concept of "blinding" presented in this manuscript is broader and includes a number of steps that may be referred to by other terms in other references may be useful.

Our objective was to explore blinding at four stages in the experiment, the first one being during the allocation and intervention. We described that “*this includes all steps of the experiment where the animals are assigned to experimental groups and the steps when they receive the experimental intervention*”. The approach of combining allocation and intervention was chosen to reflect the reality and diversity of in vivo experiments, where the point of allocation to a group and the time(s) at which animals receive interventions may be grouped together or spread out in time. Our goal was to be explicit about what stage of the experiment we referred to.

According to the Cochrane common terminology webpage, studies can be single, double or triple blinded [https://community.cochrane.org/style-manual/names-common-terms-and-terminology/common-terms-and-terminology]. Therefore those familiar with the Cochrane terminology will be comfortable that blinding should be considered in several contexts. Cochrane does note that the terms are used inconsistently [https://handbook-5-1.cochrane.org/chapter_8/8_11_2_assessing_risk_of_bias_in_relation_to_adequate_or.htm].

The Cochrane terminology is also tailored to clinical trials where the randomisation sequence is generated before subjects are gradually recruited into a trial. Allocation concealment refers to concealing the allocation sequence until treatment assignment which is a clearly defined step. In the context of animal studies however, all animals often enter the study at the same time and when a formal randomisation sequence is used, animals are randomised to experimental groups as part of the process rather than entering the study sequentially. In this context it is not clear what the time of assignment means in practice, it could refer to the time the randomisation spreadsheet is generated, or to the time the intervention is administered. The intervention is also often not a single point in time, it could be a long surgery, a series of chronic treatments administered over weeks, or a combination of both. In this context, there are no clear boundaries between allocation concealment and masking, which is why we have chosen to define masking in practical terms rather than confuse our target audience with clinical terms that have no clear equivalent in many animal studies.

To address any misunderstanding:

1. We have added a sentence in the introduction to highlight the value of being more specific as suggested by the CONSORT statement for clinical trials and the need to use an explicit terminology: “*This follows a move observed in clinical trials, where trialists are now asked to describe explicitly who was masked and how, rather than relying on ambiguous terms such as single or double masking* [17, 18]*.*”

2. In the glossary we define allocation concealment as a distinct term focused only on the assignment to treatment group up until the intervention is received.

3. We have reviewed all the use of the phrase “allocation concealment” to be sure we consistent with our language use and removed one incorrect use of the term.

11. The re-analysis of an un-blinded set of data upon a first round of "unsuccessful" tests (i.e., in the view of many, when p>0.05) is malpractice and should be called for what it is: p-hacking, accompanied by the necessary references.

Blinded analysis is a strategy to minimise bias. P hacking can be defined as “occurs when researchers collect or select data or statistical analyses until nonsignificant results become significant” [PLOS Biology 2015 Head *et al*]. The reanalysis we are advocating does not fit under this definition of p hacking as the analysis path is established prior to the analyst knowing the group identity and therefore the analytical decisions are defined without knowledge of whether things are significant or not. To ensure this misunderstanding does not arise again, we have added a sentence to be explicit that the process protects against p hacking.

“*Provided the analysis plan is then followed, p-hacking is avoided as the decisions are not being driven by whether the statistical outcome is significant or not.*“

12. I understand the need to refer to the ARRIVE at every opportunity (and the conflict of interests is duly acknowledged in the respective section), but this was submitted as a tentative academic paper and not a NC3Rs pamphlet. The constant emphasis given to reporting guidelines on what should be a matter of planning and execution to prevent unwanted errors and biases - for which there are much better guidelines and references in the literature available, most of which are not even mentioned - should make the authors pause and reflect on whether they are not tooting their own horn just a tad too much. Examples include: "The inclusion of blinding within the essential set of the ARRIVE guidelines (the Essential 10) [12], and the request to report on all four stages of an experiment is part of the journey to normalise this expectation." "The engagement of journals or professional bodies with these quality markers [the ARRIVE] will assist in the culture shift that blinding should be a standard practice."

Regarding the reviewer reflections, we have failed to find guidelines and references that do more than mention the need for blinding in animal experiments. For example, it isn’t even mentioned in the current version of PREPARE.

In the submitted manuscript, there were four references to ARRIVE.

*“Blinding is considered a gold standard to ensure the rigour and reliability of animal experiments [7, 11] and the recently updated ARRIVE guidelines, endorsed by over 1000 journals, now explicitly prompts researchers to report whether blinding was used at the different stages of the experiment – during the allocation of animals to groups and performing the experimental interventions, the conduct of the experiment (general care and welfare management), the outcome assessment, and the data analysis [12, 13].”*

*“This is corroborated by our experience during user testing of the ARRIVE guidelines 2.0, with researchers often indicating their reluctance to disclose the blinding status in their manuscript if blinding had not been used in their experiments [12].“*

*“In the ARRIVE guidelines, blinding during the conduct of the experiment refers to the handling and welfare management of the animals at any point during the experiment [13].”*

*“The inclusion of blinding within the essential set of the ARRIVE guidelines (the Essential 10) [12], and the request to report on all four stages of an experiment is part of the journey to normalise this expectation.”*

We have reviewed each use, and reworded the third and fourth sentences and left the link to ARRIVE in the references as ARRIVE and its Explanation and Elaboration are the only references which describe in detail the specific stages of an animal experiment that should be masked:

*“. Masking during the conduct of the experiment refers to the handling and welfare management of the animals at any point during the experiment [16].”*

*“The inclusion of masking within planning and reporting guidelines [10, 15, 51, 52] and the request to report on all four stages of an experiment is part of the journey to normalise this expectation.”*

13. I do not have access to the raw data, but I expect it to be available (duly anonymized).

We have addressed this point in response to reviewer 1 question 4.

14. Minor issues:

The minor issues have been corrected as suggested

* Change "For most the studies" to "For most of the studies" Done
* Change "consider oncology studies" to "considering oncology studies" Done
* The authors refer to a "Culture of Care", yet there is no reference directing readers to understand what this is about. Done
* Where the authors refer to the Irwin behavioural screening assay, a reference on this assay should be added. Done
* I would suggest in table 4 to divide the third column (Strengths and weaknesses) into two columns, or each cell divided longitudinally into "strengths"/"weaknesses") Done

Yours sincerely,

Natasha Karp on behalf of the author team.
